# Mental health services use and depressive symptom scores among gay and bisexual men in Canada

**DOI:** 10.1007/s00127-022-02362-3

**Published:** 2022-09-19

**Authors:** Ivan Marbaniang, Eric Rose, Erica E. M. Moodie, Trevor A. Hart, Joseph Cox

**Affiliations:** 1 Department of Epidemiology, 2001 McGill University, Suite 1200, Montreal, QC H3A 1G1 Canada; 2 Department of Biostatistics, McGill University, Montreal, QC Canada; 3 Department of Epidemiology, Biostatistics and Occupational Health, McGill University, Montreal, QC Canada; 4 Department of Psychology, Ryerson University, Toronto, ON Canada; 5 Dalla Lana School of Public Health, University of Toronto, Toronto, ON Canada; 6 Direction Régionale de Santé Publique de Montréal, Montreal, QC Canada; 7 Clinical Outcomes Research and Evaluation, Research Institute–McGill University Health Centre, Montreal, QC Canada

**Keywords:** Mental health services, Depression, Men’s health, Sexual and gender minorities

## Abstract

**Purpose:**

To evaluate the association between mental health services (MHS) use and depressive symptom scores among gay and bisexual men (GBM) and compare with heterosexual men in Canada.

**Methods:**

We used data from the 2015–2016 cycles of the Canadian Community Health Survey. Depressive symptoms were assessed using the PHQ-9 questionnaire (prior two weeks). MHS consultations with any licensed mental health professional (prior year) were categorized as 0, 1, 2–11, ≥ 12. We fit linear regression models to quantify the associations between MHS use and PHQ-9 scores, with an interaction term for sexual identity (GBM and heterosexual men). Models were adjusted for socioeconomic and health-related indicators.

**Results:**

Among 21,383 men, 97.3% self-identified as heterosexual and 2.7% as GBM. Compared to heterosexual men, GBM used any MHS (21% vs. 10%, *p* < 0.05) and consulted ≥ 2 health professionals for their mental health (6% vs. 2%, *p* < 0.05) in the preceding year more frequently. Overall, mean PHQ-9 scores were higher among GBM compared to heterosexual men (3.9 vs. 2.3, *p* < 0.05). Relative to no consultations, higher MHS use (2–11, ≥ 12 consultations) was associated with higher PHQ-9 scores (1.4–4.9 points higher). Associations between MHS use and PHQ-9 scores did not differ statistically between GBM and heterosexual men.

**Conclusion:**

Our findings were inconclusive in demonstrating a difference between heterosexual men and GBM for the association between MHS use and PHQ-9 scores. However, GBM consistently had higher average PHQ-9 scores for every category of consultations. Considering the higher use of MHS and higher burden of depressive symptoms among GBM, more research is needed.

**Supplementary Information:**

The online version contains supplementary material available at 10.1007/s00127-022-02362-3.

## Introduction

Increasing evidence indicates that gay and bisexual men (GBM) use mental health services (MHS) more frequently than heterosexual men [1–3]. This is despite the historical lack of a sexual-minority affirmative stance of many MHS providers and the continued practice of conversion therapy by a minority of these providers [4]. This paradox can be explained by the greater MHS need of GBM, consistent with the higher burden of poor mental health among GBM compared to their heterosexual counterparts [5, 6]. Moreover, it has been observed that GBM use MHS at levels comparable to lesbian, queer, and bisexual women, effectively closing the gender gap that exists for MHS use between heterosexual women (high use) and men (low use) [1]. Most studies in the general population have not reported on the association between MHS use and mental health by sexual identity, impeding the extrapolation of study findings to GBM [7]. Conversely, studies comprised exclusively of GBM have mostly evaluated MHS in the context of factors associated with use or unmet MHS needs [8–10]. At present, there is limited understanding of the association between MHS use and the mental health of GBM [11].

Depression is one of the most common mental health conditions reported among GBM [12]. Previous estimates from a meta-analysis using data from over 10,000 men showed that the prevalence of depression among GBM was more than twice that in heterosexual men [13]. Further, a recent modeling study using survey data from the US found that the prevalence and morbidity burden of depressive episodes in GBM have superseded that of HIV (a leading cause of morbidity among GBM) [14]. A public health concern in itself, depression among GBM is also associated with increased suicidality, substance use disorders, risky sexual behaviors and the acquisition of HIV and other sexually transmitted infections [6]. Thus, for GBM accessing MHS, these services may be instrumental not only in modifying depressive symptoms, but their use may also have associations with conditions/behaviors distally related to the symptoms.

In Canada, up to 80% of MHS have conventionally been provided by family physicians who use pharmacotherapy as a predominant treatment modality [15, 16]. Two provinces in the country (Ontario and Quebec) have recently committed to increase the delivery of publicly funded evidence-based psychotherapy for depression and anxiety, consistent with the United Kingdom’s Improving Access to Psychological Therapies (IAPT) model [17]. However, even though Canadian GBM have been identified as a group at risk for depression [12], neither of the two provinces have elucidated plans to adapt the proposed psychological services for GBM. Therefore, it is unclear if the proposed services will be successful in addressing depression among GBM beyond the existing MHS.

In this paper, we assess the association between MHS use in the Canadian health system and depressive symptoms and evaluate if the association is modified by sexual identity, that is, between GBM and heterosexual men. We frame these objectives keeping in mind the call by many countries for better assessment of MHS among GBM [7, 18].

## Methods

### Database

We used publicly available data from the 2015–2016 cycles of the Canadian Community Health Survey (CCHS). The CCHS is a nationally representative complex cross-sectional survey that collects information using computer-assisted interviewing on health status, health care utilization and health determinants across Canada’s 10 provinces and 3 territories. Its design components include stratification, multiple stages of selection and unequal probabilities of respondent selection [19].

We included data on all men ≥ 18 years of age, from 7 provinces and 1 territory where the Patient Health Questionnaire Depression Scale–9 (PHQ-9), a measure of depressive symptoms modeled on the Diagnostic and Statistical Manual-5 (DSM-5) criteria for major depressive disorder, was administered. Data from Quebec, Alberta, British Columbia, Yukon, and Nunavut, where the PHQ-9 was not administered, were excluded. Men were also excluded if they had missing data on PHQ-9 scores (8%) or sexual identity (9%).

### Study definitions

The *outcome variable* was depressive symptom scores based on the presence of related symptoms during the two preceding weeks (PHQ-9). Total PHQ-9 scores (assessed using 9 questions scored on a 0–3 Likert scale) were directly available in the dataset and ranged between 0 and 27. These were used as continuous scores, based on findings from recent literature which indicate that PHQ-9 cut-offs have a propensity to overestimate levels of depression [20].

The *explanatory variable of interest* was MHS use. This was defined as the number of mental health consultations with a licensed health professional in the preceding 12 months. Health professionals included family physicians, psychiatrists, psychologists, nurses, and social workers. The number of consultations was categorized as 0, 1, 2–11, and ≥ 12 consultations. This was done according to the observed frequency distribution wherein there was a high aggregation of individuals with 1 consultation and ≥ 12 consultations, and a sparse number of individuals with 2–11 consultations. Categorizing MHS use in this manner also allowed us to assume a non-monotonic relationship between the explanatory variable of interest and outcome i.e., every unit increase in the number of mental health consultations is not considered equivalent.

We treated the self-reported sexual identity of participants as an *effect modifier* of the relationship between the outcome variable and the explanatory variable of interest. Gay and bisexual men were grouped together (GBM), and heterosexual men constituted the comparison group.

Covariates included variables shown or strongly hypothesized to act as possible confounders, by being predictors of both MHS use and depressive symptoms, and which were available in the CCHS dataset. These included age, racial identity, presence (or absence) of a clinically diagnosed chronic mental health condition, annual household income, personal educational attainment, having a regular healthcare provider, marital status, living arrangement and heavy drinking. We categorized these covariates as presented in Table 1 and have provided details in the supplementary section on how these were collected and reported in the CCHS.

**Table 1:** Characteristics of self-identified gay/bisexual and heterosexual men from the 2015–2016 CCHS cycles (weighted estimates)

	Total (*n* = 6,326,364) (%, 95% CI)	Gay or bisexual (*n* = 173,394) (%, 95% CI)	Heterosexual (*n* = 6,152,970) (%, 95% CI)
**Total**^a^	–	97.3 (96.7–97.7)	2.7 (2.3–3.2)
**Age groups** (years)*			
18–29	21.1 (20.1–21.9)	36.9 (28.7–45.9)	20.7 (19.9–21.5)
30–39	16.5 (16.0–17.9)	13.7 (9.5–19.4)	17.0 (16.1–18.0)
40–49	18.1 (17.4–18.8)	13.8 (9.1–20.4)	18.2 (17.5–18.9)
50–59	19.1 (18.5–19.7)	15.4 (10.0–22.9)	19.2 (18.6–19.7)
60–69	14.9 (14.2–15.6)	12.9 (7.6–21.2)	14.9 (14.2–15.6)
≥ 70	9.8 (9.4–10.3)	7.3 (4.0–12.9)	9.9 (9.5–10.4)
**Personal educational attainment**			
< Secondary school	10.6 (9.9–11.2)	4.7 (2.8–7.8)	10.7 (10.1–11.4)
Secondary & < post-secondary	24.9 (23.8–26.0)	25.5 (18.1–34.7)	24.9 (23.7–26.0)
≥ Post-secondary	64.5 (63.3–65.7)	69.7 (60.6–77.5)	64.4 (63.2–65.6)
**Annual household income**			
< $40,000	17.7 (16.7–18.7)	22.4 (16.8–29.1)	17.5 (16.5–18.6)
$40,000–80,000	29.1 (27.9–30.2)	28.6 (21.0–37.5)	29.1 (27.9–30.2)
≥ $80,000	53.3 (52.0–54.5)	49.0 (40.1–58.0)	53.4 (52.1–54.6)
**Racial identity**^b^			
White	76.0 (74.8–77.2	80.0 (72.3–86.0)	75.9 (74.7–77.1)
Racialized	23.9 (22.–25.2)	19.9 (13.9–27.7)	24.1 (22.9–25.3)
**Immigration status**			
Canadian born	71.9 (70.8–73.1)	78.0 (70.2–84.3)	71.8 (70.6–72.9)
Immigrant	28.0 (26.9–29.2)	21.9 (15.7–	28.2 (27.0–29.4)
Marital status*			
Single	27.3 (26.3–28.2)	59.2 (50.4–67.4)	26.4 (25.4–27.4)
Married or common-law	64.5 (63.3–65.7)	33.9 (26.3–42.4)	65.4 (64.2–66.6)
Widowed/Divorced/Separated	8.2 (7.5–8.9)	6.9 (4.6–10.3)	8.2 (7.6–8.9)
**Living arrangement**^c^*			
Living alone	13.3 (11.9–14.7)	27.4 (21.4–34.3)	12.9 (11.6–14.2)
Living with others	86.7 (85.3–88.0)	72.6 (65.7–78.6)	87.1 (85.7–88.4)
**Region**^d^*			
Atlantic	13.2 (13.0–13.4)	12.8 (9.5–16.9)	13.2 (13.0–13.4)
Ontario	73.9 (73.6–74.2)	80.3 (75.4–84.4)	73.8 (73.4–74.1)
Prairies	12.6 (12.4–12.8)	6.7 (4.7–9.5)	12.8 (12.5–13.0)
Northwest Territories	0.2 (0.2–0.2)	0.2 (0.1–0.3)	0.2 (0.2–0.2)
**Regular health care provider**			
Yes	85.3 (84.3–86.2)	84.7 (77.9–89.7)	85.3 (84.3–86.2)
No	14.7 (13.8–15.7)	15.3 (10.2–22.1)	14.7 (13.8–15.7)
**Insurance covers all or part of medication cost**			
Yes	76.6 (75.4–77.7)	71.9 (61.9–80.1)	76.7 (75.6–77.8)
No	23.4 (22.3–24.6)	28.1 (19.9–38.1)	23.3 (22.2–24.4)
**Clinician diagnosed chronic mental health condition**^e*^			
Yes	9.4 (8.7–10.1)	20.5 (15.3–27.0)	9.1 (8.4–9.8)
No	90.6 (89.9–91.2	79.5 (73.0–84.7)	90.9 (90.2–91.6)
**Heavy drinking in the past 1-year**^f^*			
Yes	27.2 (26.1–28.2)	36.2 (28.1–45.2)	26.9 (25.9–27.9)
No	72.8 (71.7–73.9)	63.8 (54.8–71.9)	73.1 (72.0–74.1)
Mental health consultations in the past 12 months*			
No consultation	89.7 (88.9–90.5)	78.6 (72.2–83.8)	90.0 (89.2–90.8)
1 consultation	2.5 (2.2–2.9)	3.0 (1.6–5.6)	2.5 (2.2–2.9)
2 11 consultations	6.2 (5.6–6.8)	12.4 (8.6–17.5)	5.9 (5.4–6.6)
≥ 12 consultations	1.6 (1.3–1.9)	6.0 (3.2–10.8)	1.5 (1.2–1.8)
**Number of health professionals contacted in the past year for mental health**^g^*			
0–1	98.1(97.7–98.4)	94.2(90.0–96.7)	98.2(97.8–98)
≥ 2	1.9 (1.2–2.3)	5.8(3.3–9.9)	1.8(1.5–2.2)
Mean PHQ-9*	2.41 (2.32–2.49)	3.96 (3.30–4.61)	2.36 (2.28–2.44)

95% CIs based on 1000 replicate bootstrap weights provided in the CCHS

^*^p value for corrected survey design testing independence of groups (gay/bisexual and heterosexual men) < 0.05

^a^The total numbers for the categories below may differ, however the missingness for each varies between 0% (age, region) and 5.1% (racial identity), and do not considerably change the proportions presented

^b^The racialized group includes Aboriginal peoples men and other men belonging to ethnically minority groups (visible minority), defined as per the Canadian federal Employment Equity Act. The term racialized is used as specified in a 2016 report of the Mental Health Commission of Canada, “The Case for Diversity: Building the Case to Improve Mental Health Services for Immigrant, Refugee, Ethno-cultural and Racialized Populations"

^c^Living alone includes unattached individuals living alone; living with others includes individuals that live with other people (including unattached people living with others)

^d^Atlantic includes the provinces of Newfoundland and Labrador, Prince Edward Island, Nova Scotia & New Brunswick; Prairies include Manitoba & Saskatchewan

^e^Participants who reported to have a health professional-diagnosed mood and/or anxiety disorder expected to last or which has/have lasted for ≥ 6 months

^f^Heavy drinking: more than 5 drinks on one occasion at least once a month every month for the past one year defined as per Statistics Canada, “no” also includes teetotalers

^g^Health professional includes any of the following family physician, psychologist, psychiatrist, nurse, social worker

### Statistical analyses

We used linear regression to estimate the association between MHS use and PHQ-9 scores. We fit an interaction term between MHS use and sexual identity (heterosexual or GBM) to quantify any effect modification. Effect modification results were structured according to the recommendations of VanderWeele and Knol [21]. We used Statistics Canada sampling and replicate bootstrap weights to account for the complex sampling design and to obtain corrected standard errors [19]. Adjusted coefficients were estimated by including all covariates (specified under study definitions) in the regression model. Standardized coefficients were obtained by running the regression models on PHQ-9 scores that had been mean-centered and divided by the standard error of the PHQ-9 scores distribution.

We performed several sensitivity analyses to assess the robustness of our primary findings. In particular, we explored the robustness of our findings to the possibility that: (1) baseline levels of depressive symptoms, that is, PHQ-9 scores from one year prior, may differ between GBM and heterosexual men; (2) the linearity assumption in our regression model was incorrect; (3) excluding approximately 9% of the sample due to missing data on sexual identity affected our conclusions; (4) restricting the analysis to only respondents from Ontario since most (74%) of the analytical sample was derived from this province, and including an additional covariate (substance use, which was measured in CCHS only for Ontario) affected our conclusions. First, to account for baseline unmeasured PHQ-9 as a covariate, we used Monte Carlo simulations. The methodology used is described in the supplementary files. Second, as the range of PHQ-9 scores is bounded (i.e., 0–27), we compared our findings with estimates from tobit regression models. In these models, the coefficients are interpreted as linear estimates on latent unbounded PHQ-9 scores. Third, to account for the possibility that excluding men with missing data on sexual identity affected our estimates, we performed the analysis using “extreme case” imputations, first imputing all those with missing data as heterosexual, then imputing all missing values as GBM. The estimates under these two extreme imputation scenarios provide bounds on the observed estimate assuming a complete-data setting. Fourth, restricting the analysis to Ontario and including substance use as an additional covariate, simultaneously reduced the heterogeneity in the sample and increased available information (albeit at the cost of a smaller, less generalizable sample). Analyses were performed using Stata 16.1 and R version 4.0.2.

While we have refrained from making direct causal interpretations of our results, we attempt to frame our findings in a causal framework, by making several assumptions. We do this to enable the formulation of etiological hypotheses using cross-sectional data, facilitated by both MHS use (past year) and PHQ-9 (past 2 weeks) having defined time-bounds. First, we assumed that MHS use in the preceding 12 months was antecedent to PHQ-9 scores, which were assessed over the two weeks preceding administration of the CCHS questionnaire. Second, we assumed there was negligible MHS use in the assessment time frame of the PHQ-9. Third, we assumed that the covariates were invariant for the 12-month period between MHS use and PHQ-9 assessment and treat them as confounders. Given the relatively stable nature of the variables selected as covariates for the assessment duration (12 months), we believe this assumption is reasonable. Finally, we assumed that the covariates preceded MHS use. The first two assumptions were made to address the possibility of reverse causation and the latter two to align covariates used to meet the criteria for confounding.

### Ethics approval

As the dataset used was publicly available and had no personal identifiers, no ethical review was required as specified in the Tri-Council Policy Statement: Ethical Conduct for Research Involving Humans (TCPS 2), Government of Canada [22].

## Results

**Fig. 1:**
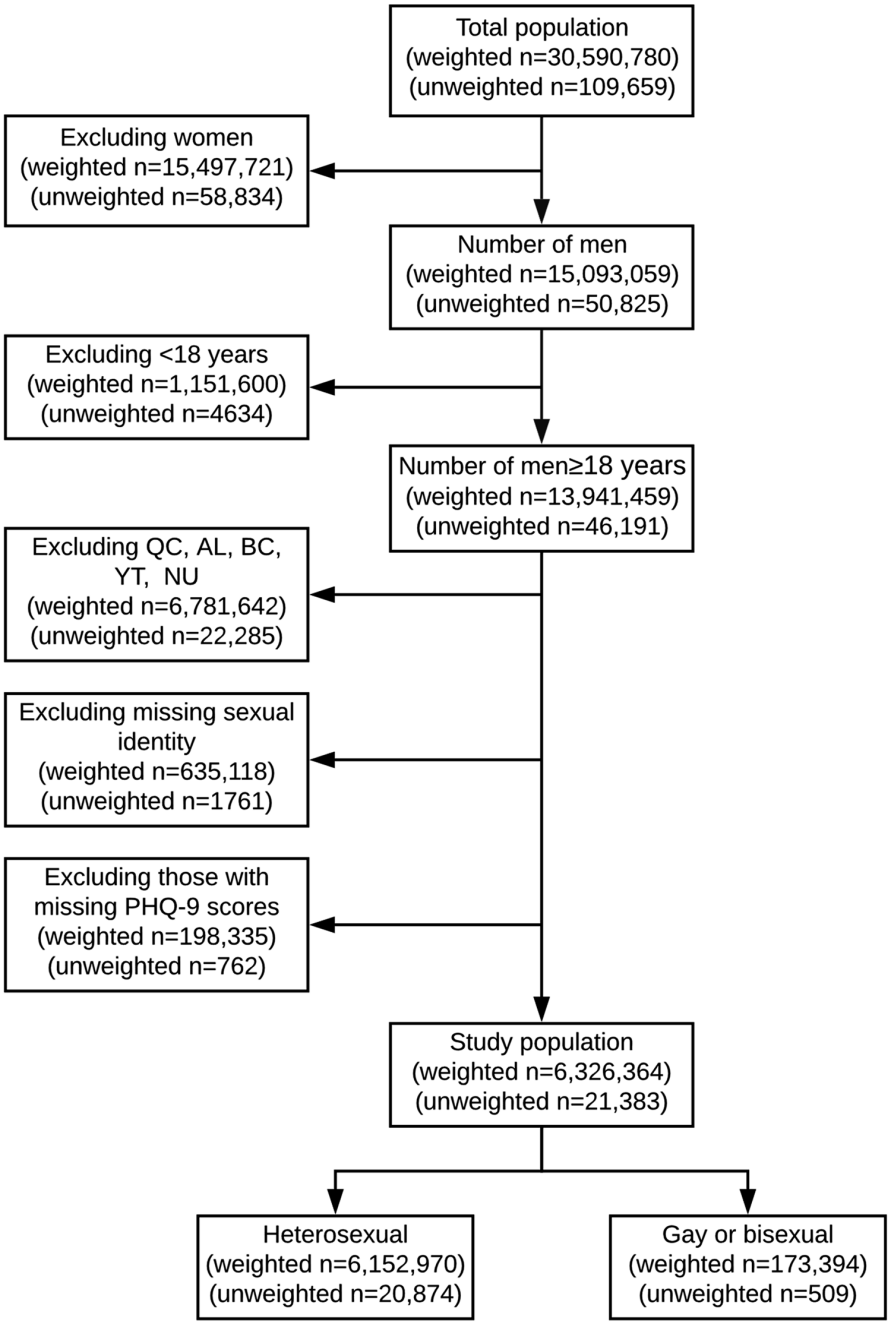
Schematic representation of the analytical population

The CCHS 2015–2016 cycles include data from 109,659 respondents representing 30,590,780 Canadians. After excluding women and those < 18 years of age, there were 46,191 (unweighted sample) respondents representing 13,941,459 (weighted sample) adult Canadian men. Our analytic sample was restricted to 21,383 respondents representing 6,326,364 adult men with available PHQ-9 scores, who self-reported their sexual identity from Ontario, Newfoundland and Labrador, Prince Edward Island, Nova Scotia, New Brunswick, Manitoba, Saskatchewan, and the Northwest Territories (excluding respondents from Quebec, Alberta, British Columbia, Yukon, and Nunavut, where the PHQ-9 was not administered). Of these, 509 respondents identified as GBM and 20,874 as heterosexual. These represented 173,394 GBM and 6,152,970 heterosexual men, respectively. The numbers of participants excluded by exclusion criteria are presented in Fig. 1.

### Description of the study sample

Overall, men < 40 years of age constituted more than a third (38%) of the study sample. Most resided in Ontario (74%), and racialized men constituted approximately a fourth (24%) of the study sample. Sixty-five percent had a post-secondary education and 53% had an annual household income of at least $80,000. Roughly one-tenth had a diagnosed chronic mental health condition or had sought at least one mental health consultation in the preceding year. Family physicians provided 57% of all mental health consultations.

**Fig. 2:**
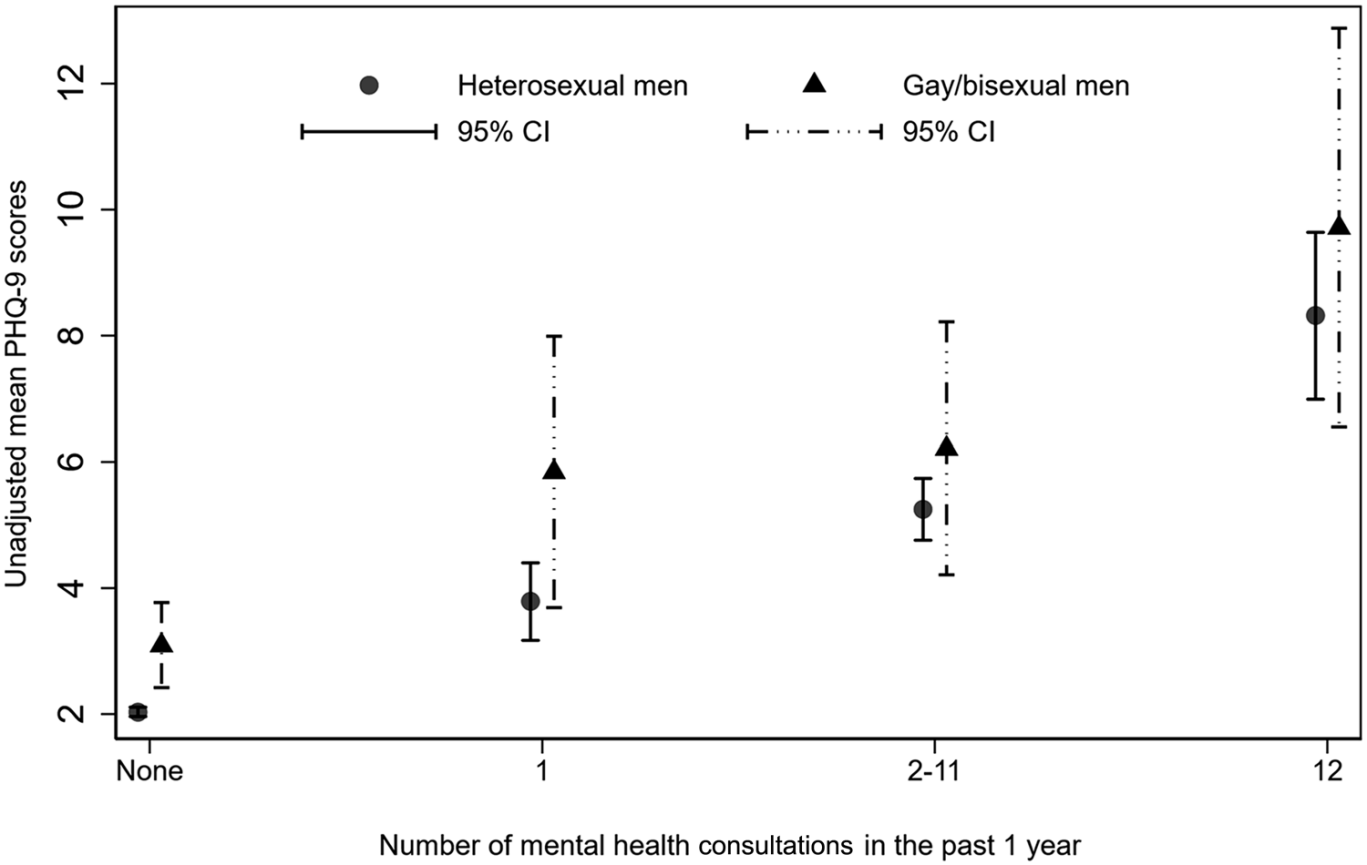
Unadjusted mean PHQ-9 scores over the number of mental health consultations by sexual identity

Compared to heterosexual men, GBM appeared to be younger, more likely to be single, living alone and residents of Ontario (*p* < 0.05). Annual household income and personal educational qualifications did not differ significantly between GBM and heterosexual men. The overall mean PHQ-9 score of GBM was significantly higher than heterosexual men (4.0, 95% CI: 3.3, 4.6 vs. 2.4, 95% CI: 2.3, 2.5; *p* < 0.05). Mean PHQ-9 scores also remained higher among GBM, with any number of mental health consultations, albeit non-significantly (Fig. 2). GBM had a significantly higher burden of chronic mental health conditions (21% vs. 9% in heterosexual men, *p* < 0.05); sought more mental health consultations (21% vs. 10%, *p* < 0.05) and consulted more than one mental health professional (6% versus 2%, *p* < 0.05) in the preceding year. (Table 1**)**.

### Linear regression model estimates

In the unadjusted model, compared to heterosexual men with no mental health consultations, mean PHQ-9 scores among GBM were 1.1 points (95% CI: 0.3, 1.8), 3.8 points (95% CI: 1.6, 6.0), 4.2 points (95% CI: 2.2, 6.1), and 7.7 points (95% CI: 4.6, 10.8) higher for no consultations, 1, 2–11 and ≥ 12 mental health consultations, respectively (Table 2). Similarly, among heterosexual men, relative to those with no consultations, mean PHQ-9 scores were 1.8 points (95% CI: 1.2, 2.4), 3.2 points (95% CI: 2.7, 3.7) and 6.3 points (95% CI: 5.0, 7.6) higher for 1, 2–11 and ≥ 12 consultations, respectively.

**Table 2:** Linear regression estimates for the relationship between mental health consultations with a health professional and PHQ-9 scores among heterosexual and gay/bisexual men

	Unadjusted model		Adjusted model*	
	Heterosexual men	Gay/bisexual men	Heterosexual men	Gay/bisexual men
	Coefficient (95% CI)	Coefficient (95% CI)	Coefficient (95% CI)	Coefficient (95% CI)
**Number of MH consultations**				
No consultations	Ref	1.06 (0.27, 1.85)	Ref	0.85 (0.10, 1.59)
1 consultation	1.75 (1.15, 2.35)	3.81 (1.65, 5.98)	0.97 (0.43, 1.51)	2.53 (- 0.52, 5.58)^a^
2–11 consultations	3.22 (2.74, 3.71)	4.18 (2.22, 6.13)	1.40 (0.91, 1.89)	1.73 (0.10, 3.53)^a^
≥ 12 consultations	6.29 (4.95, 7.63)	7.68 (4.56, 10.80)	4.15 (2.90, 5.40)	4.87 (1.42, 8.32)^a^

*MH* Mental health

*Adjusted for age, racial identity, diagnosis of a chronic mental health condition, annual household income, personal education, possession of a regular healthcare provider, marital status, living arrangement and heavy drinking

^a^For the same number of consultations, the mean values of PHQ-9 scores between gay/bisexual and heterosexual men, when adjusted for the covariate set listed above were not significantly different (interaction terms *p* > 0.1 for all categories of consultations)

When adjusted for age, racial identity, presence (or absence) of a clinically diagnosed chronic mental health condition, annual household income, personal education, having a regular healthcare provider, marital status, living arrangement, and heavy drinking, the magnitude of the associations decreased overall. In the adjusted model, compared to heterosexual men with no mental health consultations in the preceding year, mean PHQ-9 scores among GBM, were 0.8 points higher for no consultation (95% CI: 0.1, 1.6), 1.7 points higher for 2–11 consultations (95% CI: 0.1, 3.5) and 4.9 points higher for ≥ 12 consultations (95% CI: 1.4, 8.3). These corresponded to 0.2 (95% CI: 0.03, 0.4), 0.5 (95% CI: 0.03, 0.9) and 1.3 (95% CI: 0.4, 2.2) standardized points, respectively. Adjusted estimates were not significant for a single consultation (coefficient: 2.5, 95% CI: -0.5, 5.6) (Table 2).

Similarly, among heterosexual men, adjusted mean PHQ-9 scores were higher with increasing number of consultations: 1.0 point (95% CI: 0.4, 1.5) higher for a single consultation, 1.4 (95% CI: 0.9, 1.9) points higher for 2–11 consultations, and 4.2 (95% CI: 2.9, 5.4) points higher for ≥ 12 consultations, when compared to no consultations. These corresponded to 0.3 (95% CI: 0.1, 0.4), 0.4 (95% CI: 0.2, 0.5) and 1.1 (95% CI: 0.8, 1.4) standardized points, respectively.

We found no significant differences in the adjusted coefficients of MHS use on PHQ-9 scores between GBM and heterosexual men, for the same number of mental health consultations. (Table 2).

### Sensitivity analyses

When we account for baseline PHQ-9 scores, both unadjusted and adjusted point estimates are reduced overall, but patterns in the point estimates are similar to when adjustment for baseline PHQ-9 is excluded (supplementary Table 1): coefficients increase with increasing consultations and estimates for GBM are higher than for heterosexual men. This suggests that the higher symptoms among those with more MHS use is not simply a phenomenon of having had higher baseline PHQ-9 from 1 year prior. Therefore, not having measured prior PHQ-9 is not introducing bias in the estimates. As is typical with simulation-based sensitivity analyses, uncertainty is increased and confidence intervals are wider, encompassing null effects. Adjusted tobit regression estimates were comparable to the estimates from linear regression (supplementary Table 2). We present linear regression estimates as our primary findings, to make our results easier to interpret. Interpretations for the adjusted point estimates did not differ qualitatively when all those with missing sexual identity were assumed to be either GBM, or heterosexual (supplementary Table 3). Lastly, when restricting our analytical population to only include respondents from Ontario with an additional covariate for lifetime substance use, coefficients increased or decreased between 2 and 40%, but confidence intervals largely overlapped with the primary model (supplementary Table 4). This suggests that our results are mostly driven by data from Ontario. However, these results also suggest that our main findings are robust to the exclusion of lifetime substance use as a covariate.

## Discussion

Using data from a Canadian community-based survey of 21,383 men, we found that a higher number of mental health consultations were associated with higher average depressive symptom scores in both GBM and heterosexual men. Additionally, associations were not statistically different across sexual identity groups, despite GBM consistently having higher depressive symptom scores at all levels of MHS use. To our knowledge, this is the first analysis to use nationwide survey data from Canada to address a crucial research gap in understanding the association between MHS use and depressive symptom scores in GBM.

The higher average depressive symptom scores with more consultations could indicate that existing MHS are sub-optimal in mitigating depressive symptoms in all men with a greater need for MHS. As seen in our sensitivity analysis, this trend persists for both groups of men, albeit attenuated in magnitude, when adjustments for baseline PHQ-9 are made or men are assumed to start from similar PHQ-9 scores a year prior. Our findings suggest that for recurrent MHS users, changes that occur in depressive symptoms between consultations may better explain the severity of subsequent depressive symptoms than baseline depressive scores [23]. Previous work has suggested that recurrent treatment-seeking behavior is implicated in poorer treatment response [24]; a limitation of our sensitivity analysis is that we were unable to account for *time-varying* depressive symptom scores (occurring between consultations) which may confound the relationship between frequency of MHS use and subsequent depressive symptoms. This may be particularly important to address in future analyses to plan better MHS for GBM, who as our findings indicate, are more likely to exhibit recurrent MHS seeking behavior.

We observed that the association between MHS use and depressive symptom scores was non-differential by sexual identity. Our findings are consistent with findings from an evaluation of the aforementioned IAPT in the UK, in which gay but not bisexual men were found to have comparable average depressive symptom scores to heterosexual men [25]. For our data, given that GBM used proportionately more MHS than heterosexual men, we might have expected average depressive symptom scores among GBM to be lower. This assumes health professionals are familiar with the mental health needs of GBM and therefore more adept at providing MHS for them. However, as research over the past two decades indicates, explicit teaching on sexual minorities health has not achieved widespread curricular integration in health professional training [26–28], which may have limited health professionals from providing context specific and sexual identity affirming MHS [29]. Furthermore, we also observe that GBM consistently had higher average depressive symptom scores for any number of mental health consultations. Therefore, we interpret the statistically non-significant difference in our associations between the sexual identity groups cautiously, as it is likely that we are not adequately powered to detect a significant difference. Based on our findings, we are unable to draw any conclusive inferences on whether the association between MHS use and depressive symptom scores are indeed similar between the two sexual identity groups. We believe that this is an important topic worthy of further study.

There are several limitations to our study findings. Given the cross-sectional nature of survey data, we are unable to explore the mechanisms for our observations. Nonetheless, by basing our hypotheses on existing literature, using a causal framework and appropriate sensitivity analyses, we are able to posit suppositions that can be further tested in future. Additionally, the use of survey data allowed us to explicitly set up comparisons between GBM and heterosexual men. We grouped different types of MHS providers together, reducing the heterogeneity of MHS that they provide. Different types of providers and treatment modalities they employ could be differentially associated with PHQ-9 scores. However, in this manuscript we position MHS from the larger perspective of the curricula in which mental health professionals receive their training, which consistently lacks in its focus on mental health issues of sexual minorities across disciplines (Medicine, Nursing, Social Work) [26–28]. Similarly, we combined GBM into a single group cognizant that in doing so, we obfuscate the differences in mental health needs and MHS use patterns between them. However, we were not adequately powered to make meaningful distinctions in our findings when gay and bisexual men were separated i.e., interpretations of our findings remain the same as when gay and bisexual men are combined into a single group (as reported). In light of this limitation, we advocate for larger studies in future that are adequately powered to make an informative distinction in findings between gay and bisexual men. As the CCHS does not contain baseline PHQ-9 scores, we are unable to comment on the clinical significance of our findings using methods proposed by Jacobson and Traux [30]. We addressed the lack of baseline PHQ-9 scores using Monte Carlo simulations, but we refrain from making direct clinical interpretations of these findings, as simulation estimates are based on additional assumptions. Lastly, as already stated, our results may be most generalizable to Ontario.

We also make a few assumptions in our models. First, the associations observed are independent of the time when consultations occurred. This is less of a concern for a higher number of consultations, as it could be presumed that they would be distributed relatively equally over twelve months. Second, we ignore the cyclical nature of depressive symptoms [31] in our models. This should be addressed by better assessment methods (like studies using diagnostic interviews or ecological momentary assessments [32]) in future studies that can account for the cyclicity of depressive symptoms. Finally, our model fails to account for mental health disparities that exist across social hierarchies, by not being statistically powered to assess the heterogeneity of associations across racial identity, socioeconomic status or at the intersection of these social hierarchies.

Our findings highlight the importance of understanding better the inter-consultation changes in depressive symptoms, and the subsequent role that these may play in determining the effectiveness of MHS for men. We simultaneously advocate for the collection of more granular mental health data for sexual minority men accessing MHS and recommend larger comparative evaluations of MHS effectiveness between heterosexual men and GBM.

## Supplementary Information

Below is the link to the electronic supplementary material.

Supplementary file1 (DOCX 518 KB)
