# Supplementary material for: Mental health services use and depressive symptom scores among gay and bisexual men in Canada
Source: Soc Psychiatry Psychiatr Epidemiol. 2022 Sep 19;57(11):2333–42. doi: 10.1007/s00127-022-02362-3 (PMC9636296; doi:10.1007/s00127-022-02362-3)
Supplement: Supplementary file 1 — Supplementary file1 (DOCX 518 KB) [file 127_2022_2362_MOESM1_ESM.docx]

**SUPPLEMENTARY FILES**

**A**. **Questions and responses as recorded in the CCHS 2015-2016**

| **NUMBER OF MENTAL HEALTH CONSULTATIONS (explanatory variable of interest)**  In the past 12 months, that is, from [date one year ago] to yesterday, have you seen or talked to a health professional about your emotional or mental health?  How many times (in the past 12 months)?  Number of consultations with a mental health professional | Yes  No  Don't know  Refusal  Not stated  01 – 12 (If the participant responded “**Yes”** to the question above)  (Values ≥12 were capped at 12 by Statistics Canada to prevent accidental disclosure of study participants when using the publicly available dataset)  0 (If the participant answered “**No”** the question above) |
| --- | --- |
| **DEPRESSIVE SYMPTOMS SEVERITY SCORES (outcome variable)**  This variable indicates the severity of a respondent's depressive symptoms, using a 9-item scale.  This scale corresponds to the Patient  Health Questionnaire (PHQ-9). | Score obtained on the depression scale (directly available in the CCHS)  (minimum score: 0, maximum score: 27)  A higher score indicates more severe depressive symptoms. |
| **SEXUAL IDENTITY (effect modifier)**  Do you consider yourself to be? | Heterosexual  Homosexual  Bisexual  Don’t know  Refusal  Not stated |
| **AGE (covariate)**  What is your age? | Age between 18 and 19  Age between 20 and 24  Age between 25 and 29  Age between 30 and 34  Age between 35 and 39  Age between 40 and 44  Age between 45 and 49  Age between 50 and 54  Age between 55 and 59  Age between 60 and 64  Age between 65 and 69  Age between 70 and 74  Age between 75 and 79  Age 80 and older |
| **RACIAL IDENTITY (covariate)**  Cultural / racial background | White  Non-white (Aboriginal or Other Visible Minority)  Not stated |
| **CHRONIC MENTAL HEALTH CONDITION (covariate)**  Participants that reported to have a health professional diagnosed mood AND/OR anxiety disorder which has/have lasted for ≥ 6 months.  Do you have a mood disorder such as depression, bipolar disorder, mania or dysthymia?  Do you have an anxiety disorder such as a phobia, obsessive-compulsive disorder or a panic disorder? | Yes  No  Don't know  Refusal  Not stated  Yes  No  Don't know  Refusal  Not stated |
| **ANNUAL HOUSEHOLD INCOME (covariate)**  This variable groups the total household income from all sources which could be wages/salaries or self-employment and/or employment insurance or worker’s compensation or social assistance/welfare and/or Benefits from Canada or Quebec Pension Plan or job-related retirement pensions, superannuation and annuities or RRSP (Registered Retirement Savings Plan)/RRIF (Registered Retirement Income Fund) of Old Age Security and Guaranteed Income Supplement. | No income or less than $20,000  $20,000 to $39,999  $40,000 to $59,999  $60,000 to $79,999  $80,000 or more  Not answered |
| **PERSONAL EDUCATIONAL ATTAINMENT (covariate)**  This variable indicates the highest level of education attained by the respondent. | Less than secondary school graduation  Secondary school graduation, no post-secondary education  Post-secondary certificate/diploma or university degree  Don’t know, refusal, not stated |
| **REGULAR HEALTHCARE PROVIDER**  **(covariate)**  Do you have a regular health care provider? By this, we mean one health professional that you regularly see or talk to when you need care or advice for your health. | Yes  No  Don't know  Refusal  Not stated |
| **MARITAL STATUS (covariate)**  What is your marital status?  Are you? | Married  Common-law  Widowed/Divorced/Separated  Single  Not stated |
| **LIVING ARRANGEMENT (covariate)**  Living / family arrangement of selected respondent. | Unattached individual living alone  Unattached individual living with others  Individual living with spouse/partner  Parent living with spouse/partner and child(ren)  Single parent living with children  Child living with a single parent with or without siblings  Child living with two parents with or without siblings  Other  Not stated |
| **HEAVY DRINKING (covariate)**  The Statistics Canada definition for heavy drinking was used which is >=5 drinks every month or more in the last 1 year.  How often in the past 12 months have you had [5 (male) / 4 (female)] or more drinks on one occasion? | Never  Less than once a month  Once a month  2 to 3 times a month  Once a week  More than once a week  Don’t know  Refusal  Not stated |
| **DRUG USE (covariate only for ONTARIO)**  This variable indicates whether respondents used any of the drugs listed in the past 12 months. Excludes one time use of marijuana or hashish. | Has used drugs in the past 12 months (excluding marijuana "just once")  Has not used drugs 12 months (excluding marijuana "just once")  Not stated |

**B. Method used to adjust for the unmeasured baseline PHQ-9 score**

We use Monte Carlo sensitivity analysis to assess whether the conclusions of this analysis were robust to PHQ-9 scores at baseline being unobserved. That is, we assess the impact of a possible violation of the assumption of no unmeasured confounding, with baseline PHQ-9 as the potential confounder. We examine both the unadjusted and adjusted models used in the analysis.

To do this we first posit a parametric model for the mean baseline PHQ-9 scores conditional on the number of mental health consultations, sexual identity, and the strongest *measured* confounder, which is diagnosis of a chronic mental health condition. In addition, we posit a model for the mean PHQ-9 outcome score that matches the analysis model for both scenarios with an additional term for the effect of baseline PHQ-9 scores on the final PHQ-9 outcome.

Let $Y$ denote the PHQ-9 outcome score, $X_{1}$ denote an indicator for being a gay or bisexual man (GBM), $X_{2}$ denote an indicator for diagnosis of a chronic mental health condition, $A$ denote the number of mental health consultations as a factor with 4 levels, and $U$ denote the baseline PHQ-9 score. Then our posited models are given by $E\left( U | X, A; \xi\right)$ and $E\left( Y | X,A,U;\beta, \psi\right)$ such that $\psi$ denotes the parameters of interest related to the effect of mental health consultations and sexual identity on the final PHQ-9 score, $\beta$ denotes the remaining regression coefficients, $\xi$ denotes the parameters characterizing the dependence of the unmeasured confounder on the treatment and measured confounders, and *X*=( $X_{1}$, $X_{2}$).

We posit parametric distributions for $\xi$, the parameters indexing the model for the mean baseline PHQ-9 score. We will also posit a parametric distribution for $\beta_{U}$, the coefficient for the effect of baseline PHQ-9 on the PHQ-9 outcome. Monte Carlo sensitivity analysis proceeds by drawing estimates for $\hat{\xi}$ and  $\hat{\beta}_{U}$ from their respective posited distributions for each Monte Carlo repetition. We can then calculate the expected bias in the estimation of $\psi$ if $U$ is left out of our regression model for each repetition using its draw of $\hat{\xi}$ and  $\hat{\beta}_{U}$ . A point estimate for the bias adjusted value of $\psi$ is given by the average of the bias adjusted estimates. To generate confidence intervals, we calculate standard errors following how multiple imputed data sets are pooled in multiple imputation to account for the uncertainty due to both sampling variability and unmeasured confounding. Here the “within” variance represents the variance of $\hat{\psi}$ for a given draw of $\hat{\xi}$ and  $\hat{\beta}_{U}$ instead of a single imputed data set and the “between” variance represents the variance of $\hat{\psi}$ between the different Monte Carlo repetitions. We obtain the “within” variance by averaging the estimated variance from each repetition and the “between” variance by calculating the variance of all the bias adjusted estimates. Each of the variances calculated within each repetition also incorporate the replicate weights. We then use Rubin’s variance formula to get an estimate for the variance and use this to calculate 95% confidence intervals [33].

We will let $\xi$ and $\beta$ both be normally distributed. To set the mean and standard deviation of the distribution for $\xi$ we will use the final PHQ-9 score, $Y$, as a surrogate for the baseline PHQ-9 score and fit the posited regression model, $E\left( U | X, A; \xi\right)$, with $Y$ replacing $U$. The mean of the distribution of $\xi$ is then given by the estimate from the surrogate model scaled by a constant factor which we will take to be 0.3. The distribution for the intercept of the model, $\xi_{0}$, will have its mean set such that $E\left\{ E\left( U | X, A; \xi\right) \right\}=\bar{Y}$ after scaling the remaining parameters. The standard deviation for each distribution will be set to the standard error of each parameter gathered from fitting the surrogate model. The distribution of $\beta_{U}$ will be assumed to be normal with a mean of 1 and a standard deviation of 0.25. These parameter values were chosen based on a longitudinal study that showed minimal change in PHQ scores over 1 year for patients that did not receive any treatment [34].

**C. Sensitivity analyses results**

| Supplementary Table 1 (sensitivity analysis 1): Linear regression estimates for the relationship between mental health consultations with a health professional and PHQ-9 scores among heterosexual and gay/bisexual men *after accounting for unmeasured baseline PHQ-9* | | | | |  |
| --- | --- | --- | --- | --- | --- |
|  | **Unadjusted model** | | **Adjusted model*** | | |
|  | **Heterosexual men** | **Gay/bisexual men** | **Heterosexual men** | **Gay/bisexual men** | |
|  | Coefficient (95% CI) | Coefficient (95% CI) | Coefficient (95% CI) | Coefficient (95% CI) | |
| Number of MH consultations  No consultations  1 consultation  2-11 consultations  ≥12 consultations | Ref  1.23 (0.54, 1.91)  2.25 (1.57, 2.94)  4.40 (2.79, 6.01) | 0.76 (-0.08, 1.59)  2.74 (0.36, 5.13)  2.86 (0.88, 4.84)  5.24 (1.88, 8.60) | Ref  0.67 (0.05, 1.30)  1.00 (0.43, 1.57)  2.85 (1.40, 4.31) | 0.57 (-0.23, 1.38)  2.09 (-1.12, 5.30)  1.07 (-0.69, 2.83)  3.51 (-0.11, 7.13) | |
| MH – Mental health  *Adjusted for age, racial identity, diagnosis of a chronic mental health condition, annual household income, personal education, possession of a regular healthcare provider, marital status, living arrangement and heavy drinking  Note: The sensitivity analysis does not reveal large shifts in point estimates, especially for GBM. As is common in simulation-based methods, uncertainty is increased and hence 95 % CIs are wider, and encompass a null effect. | | | | | |

| Supplementary Table 2 (sensitivity analysis 2): Tobit regression estimates for the relationship between mental health consultations and PHQ-9 scores among heterosexual and gay/bisexual men | | | | |  |
| --- | --- | --- | --- | --- | --- |
|  | **Unadjusted model** | | **Adjusted model*** | | |
|  | **Heterosexual men** | **Gay/bisexual men** | **Heterosexual men** | **Gay/bisexual men** | |
|  | Coefficient (95% CI) | Coefficient (95% CI) | Coefficient (95% CI) | Coefficient (95% CI) | |
| Number of MH consultations  No visits  1 visit  2-11 visits  ≥12 visits | Ref   - 1. (1.82, 3.42)   4.57 (3.97, 5.16)  7.80 (6.38, 9.22) | 1.77 (0.74, 2.79)  5.28 (2.62, 7.94)  5.67 (3.46, 7.87)  9.32 (6.04, 12.60) | Ref  0.96 (0.40, 1.53)  1.40 (0.93, 1.88)  4.11 (2.85, 5.38) | 0.88 (0.19, 1.52)  2.66 (-0.34, 5.66)  1.75 (0.04, 3.46)  5.05 (1.38, 8.73) | |
| MH – Mental health  *Adjusted for age, racial identity, diagnosis of a chronic mental health condition, annual household income, personal education, possession of a regular healthcare provider, marital status, living arrangement and heavy drinking | | | | | |

| Supplementary Table 3 (sensitivity analysis 3): Adjusted linear regression estimates for the relationship between mental health consultations and PHQ-9 scores among heterosexual and gay/bisexual men, comparing three scenarios | | | | | | | | |
| --- | --- | --- | --- | --- | --- | --- | --- | --- |
|  | **Primary model** | | **Model assuming all those with missing sexual identity were heterosexual** | | **Model assuming all those with missing sexual identity were gay/bisexual** | | | |
|  | **Heterosexual men** | **Gay/bisexual men** | **Heterosexual men** | **Gay/bisexual men** | **Heterosexual men** | **Gay/bisexual men** | | |
|  | Coefficient (95% CI) | Coefficient (95% CI) | Coefficient (95% CI) | Coefficient (95% CI) | Coefficient (95% CI) | | | Coefficient (95% CI) |
| Number of MH consultations  No visits  1 visit  2-11 visits  ≥12 visits | Ref  0.97 (0.43, 1.51)  1.40 (0.91, 1.89)  4.15 (2.90, 5.40) | 0.85 (0.10, 1.59)  2.53 (- 0.52, 5.58)  1.73 (0.10, 3.53)  4.87 (1.42, 8.32) | Ref  0.94 (0.38, 1.50)  1.35 (0.87, 1.84)  4.18 (2.90, 5.46) | 0.84 (0.17, 1.51)  2.59 (-0.44, 5.64)  1.69 (-0.02, 3.41)  4.95 (1.26, 8.64) | Ref  0.95 (0.38, 1.52)  1.36 (0.87, 1.85)  4.06 (2.78, 5.33) | | 0.47 (-0.12, 1.07)  1.90 (-0.48, 4.29)  1.53 (0.04, 3.02)  5.89 (2.02, 9.77) | |
| MH – Mental health  *Adjusted for age, racial identity, diagnosis of a chronic mental health condition, annual household income, personal education, possession of a regular healthcare provider, marital status, living arrangement and heavy drinking | | | | | | | | |

| Supplementary Table 4 (sensitivity analysis 4): Linear regression estimates for the relationship between mental health consultations and PHQ-9 scores among heterosexual and gay/bisexual men only for Ontario | | | | |  |
| --- | --- | --- | --- | --- | --- |
|  | **Unadjusted model** | | **Adjusted model*** | | |
|  | **Heterosexual men** | **Gay/bisexual men** | **Heterosexual men** | **Gay/bisexual men** | |
|  | Coefficient (95% CI) | Coefficient (95% CI) | Coefficient (95% CI) | Coefficient (95% CI) | |
| Number of MH consultations  No visits  1 visit  2-11 visits  ≥12 visits | Ref  1.42 (0.68, 2.17)  3.01 (2.39, 3.64)  6.05 (4.36, 7.75) | 1.20 (0.23, 2.18)  3.93 (1.17, 6.69)  4.75 (2.20, 7.30)  7.01 (3.70, 10.31) | Ref  0.64 (0.02, 1.26)  1.32 (0.69, 1.96)  4.08 (2.54, 5.62) | 0.95 (0.03, 1.64)  2.92 (-0.67, 6.51)  2.42 (0.41, 4.42)  4.15 (0.52, 7.79) | |
| MH – Mental health  *Adjusted for age, racial identity, diagnosis of a chronic mental health condition, annual household income, personal education, possession of a regular healthcare provider, marital status, living arrangement, lifetime drug use (excluding single time use) and heavy drinking | | | | | |

**D: Linear regression estimates for the adjusted model including covariates**

The estimates for the final multivariate model are provided in supplementary Table 5 below. However, we refrain from interpreting the associations between the covariates and the outcome in the same manner as the association between the *explanatory variable of interest* (MHS use) and the outcome. The underlying framework for the selection of covariates in this model is causal, i.e., covariates other than MHS use are viewed as probable confounders of the MHS use/PHQ-9 relationship (i.e., these variables precede both the explanatory variable of interest and the outcome and are believed to causally affect each of these variables). As such, no attempt at controlling for possible confounding in the associations between these covariates and the outcome has been made and further, we believe that MHS use lies on the causal pathway between these variables and the outcome (i.e., MHS use acts as a mediator, and hence its inclusion in the model my distort the relationship between these variables and PHQ-9 scores). Interpreting the associations between the covariates and the outcome in the same manner as that for the exposure of interest may result in a **Table 2 fallacy** [35].

We are primarily interested in estimating the association between MHS use and PHQ-9 scores. To explain the Table 2 fallacy in this context, suppose for simplicity we identify only two confounders: living with a chronic mental health condition (i.e., living with a mental health condition is associated with higher probabilities of using MHS and reporting higher PHQ-9 scores) and age. See SFig1 for a causal graph depicting the assumed relationships.

**SFig1**:


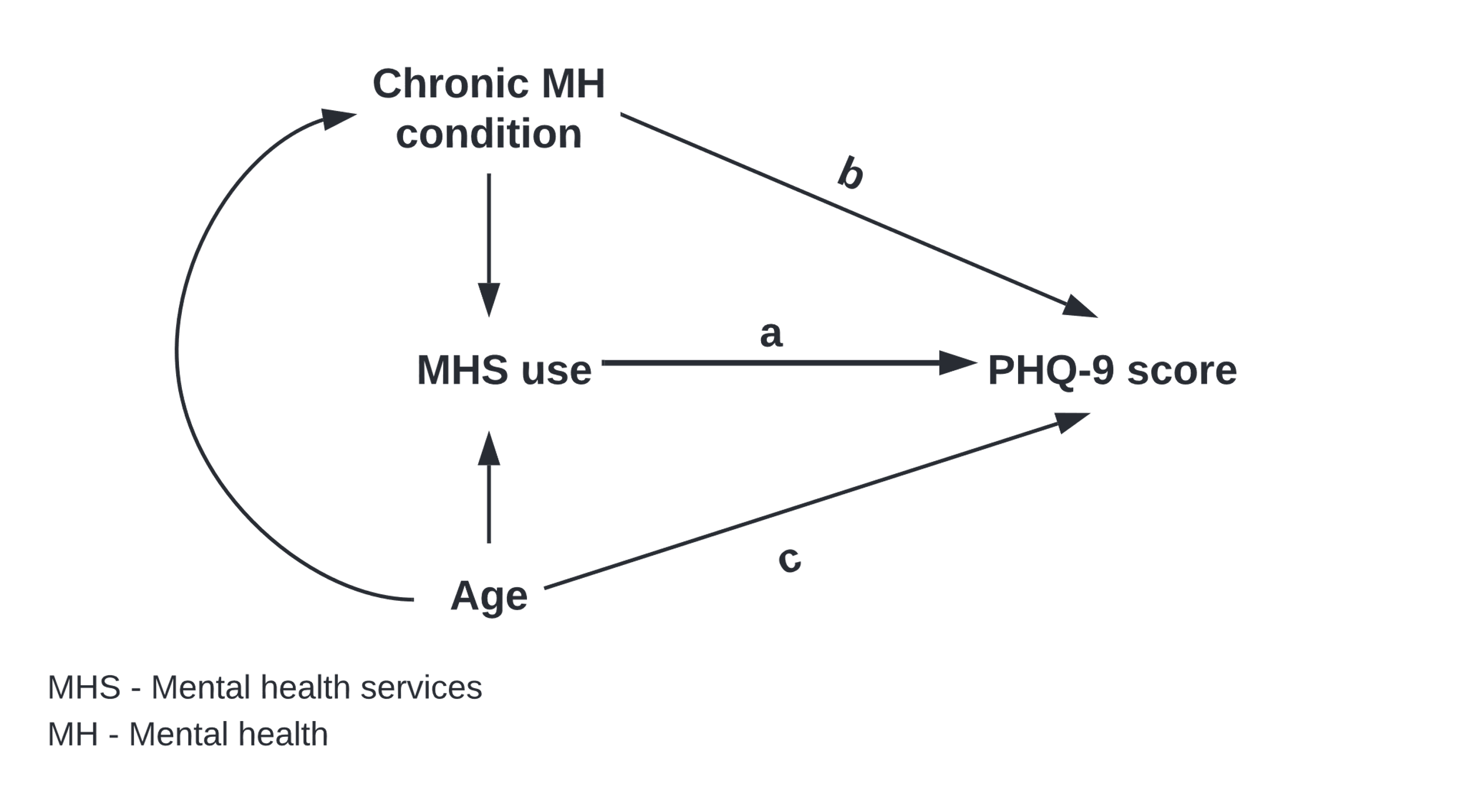


Directed acyclic graph showing the relationships between the explanatory variable of interest, confounder variables and the outcome, when there are no unmeasured confounders for the relationship between chronic MH condition and PHQ-9 score.

When we adjust/condition for the two confounders:

1. The relationship (a) shown in SFig1 can be interpreted as the association between MHS use and PHQ-9 score at any given level of age and chronic mental health condition.
2. The relationship (b) is interpreted as that portion of the association between living with a chronic mental health condition and PHQ-9 score that is not mediated through the association between living with chronic mental health condition and MHS use, assuming that there are no unmeasured confounders for the relationship (b) and linear models are appropriate [36]. A similar interpretation can be made for age, for the relationship (c).

Thus, we see that the interpretations of the associations if there are no unmeasured confounders for the relationship (a) and (b) are already different, i.e., the estimates for the explanatory variable of interest cannot be interpreted in the same manner as the estimates for a covariate.

Further, consider a situation in which there is an unmeasured confounder for relationship (b), for example a family history of having a mental illness as shown in SFig2.

**SFig2:**


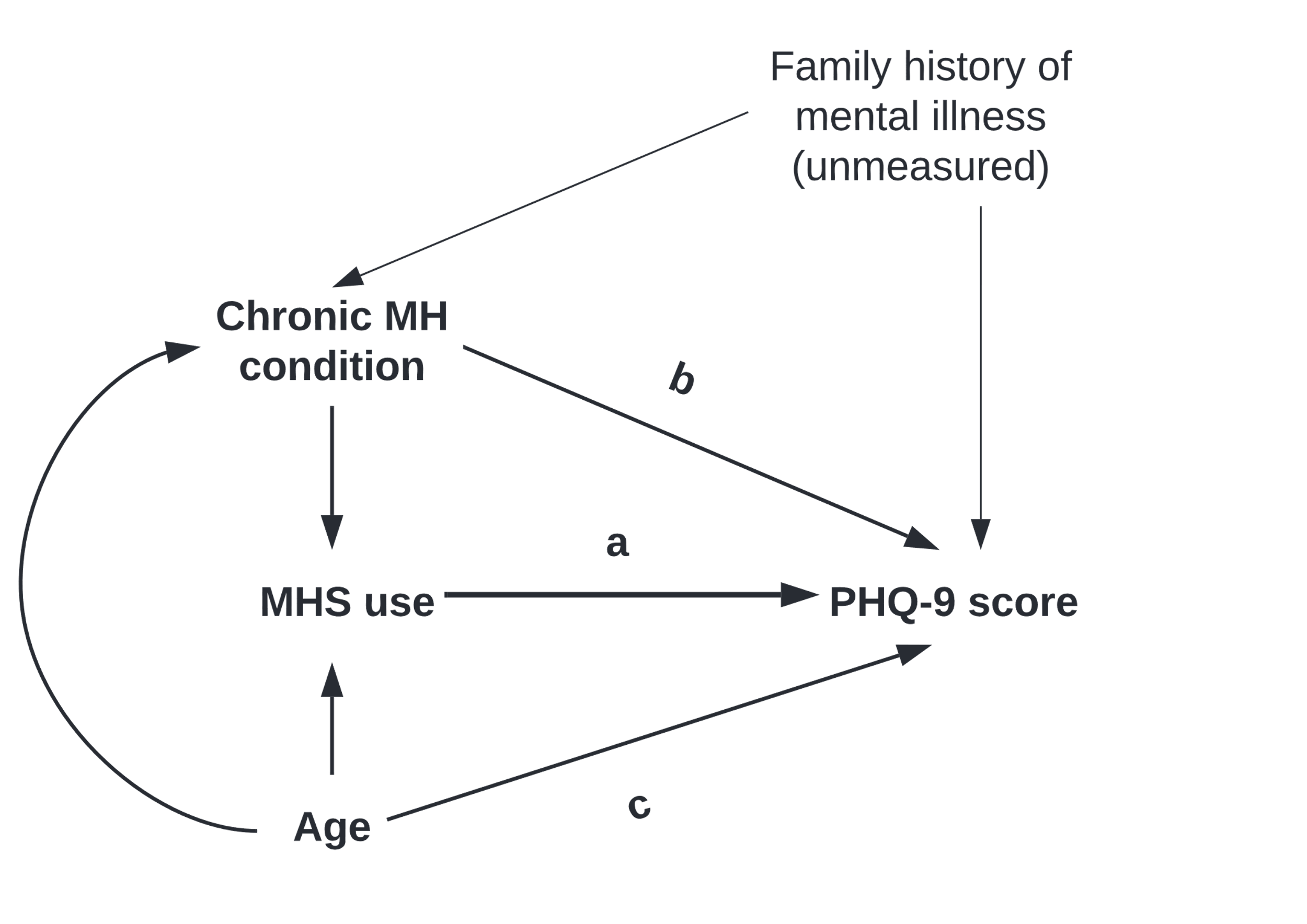


Directed acyclic graph showing the relationships between the explanatory variable of interest, confounder variables and the outcome, when there is an unmeasured confounder for the relationship between chronic MH condition and PHQ-9 score.

In this situation, when we adjust/condition for the two known confounders (living with a chronic mental health condition, age):

1. The interpretation for relationship (a) remains the same, as family history of mental illness does not affect MHS use (except through living with a chronic MH condition, but this path is blocked by adjustment for the chronic MH condition), and therefore it is not a confounder.
2. Relationship (b) can no longer be interpreted a valid estimate for that portion of the association between living with a chronic MH condition and PHQ-9 score that is not mediated through the association between living with chronic mental health condition and MHS use, because no control for the unmeasured confounder has been made.
3. Controlling for living with a chronic mental health condition also opens an indirect path from age to the unmeasured confounder and PHQ-9 score. Thus, the covariate for age is no longer interpretable as a valid estimate for that portion of the association between age and PHQ-9 scores that is not mediated through the association between age and MHS use.

| **Supplementary Table 5: Adjusted linear regression estimates for the whole model** | | |
| --- | --- | --- |
|  |  | **Adjusted model**  Coefficient (95% CI) |
| **Explanatory variable of interest** | **Number of MH consultations**  No visits  1 visit  2-11 visits  ≥12 visits | Ref  0.97 (0.43, 1.51)  1.40 (0.91, 1.89)  4.15 (2.90, 5.40) |
| **Effect modifier** | **Sexual identity**  Heterosexual  Gay/bisexual men | Ref  0.85 (0.10, 1.59) |
| **Statistical interaction term** | **Interaction term**  Heterosexual * No visits  Gay/bisexual * 1 MH visit  Gay/bisexual * 2-11 MH visits  Gay/bisexual * ≥12 MH visits | Ref  0.84 (-2.67, 4.36)  - 0.50 (-2.39, 1.38)  0.09 (-3.63, 3.81) |
| **Covariates** | **Age (in years)**  18 – 29  30 – 39  40 – 49  50 – 59  60 – 69  ≥70 | Ref  -0.27 (-0.61, 0.06)  -0.29 (-0.63, 0.05)  -0.30 (-0.62, 0.02)  -0.71 (-1.04, -0.38)  -0.74 (-1.10, -0.37) |
|  | **Living with a chronic MH condition**  No  Yes | Ref  3.53 (3.11, 3.95) |
|  | **Annual household income**  <$40,000  $40,000 - $80,000  ≥$80,000 | Ref  -0.38 (-0.60, -0.15)  -0.70 (-0.95, -0.46) |
|  | **Personal educational attainment**  <Secondary school  Secondary & <post-secondary  ≥Post-secondary | Ref  -0.18 (-0.48, 0.11)  -0.46 (-0.74, -0.18) |
|  | **Racial identity**  White  Racialized | Ref  0.13 (-0.09, 0.36) |
|  | **Regular health care provider**  No  Yes | Ref  -0.20 (-0.48, 0.07) |
|  | **Marital status**  Married or common-law  Single  Widowed/Divorced/Separated | Ref  0.27 (-0.02, 0.56)  0.44 (0.14, 0.74) |
|  | **Living arrangement**  Living with others  Living alone | Ref  -0.14 (-0.41, 0.13) |
|  | **Heavy drinking in the past 1-year**  No  Yes | Ref  0.23 (0.04, 0.42) |

**References**

33. Rubin DB. (1987) Multiple imputation for nonresponse in surveys. In: New York: Wiley.

34. Okereke, O. I., Reynolds, C. F., 3rd, Mischoulon, D., Chang, G., Vyas, C. M., Cook, N. R., Weinberg, A., Bubes, V., Copeland, T., Friedenberg, G., Lee, I. M., Buring, J. E., & Manson, J. E. (2020). Effect of Long-term Vitamin D3 Supplementation vs Placebo on Risk of Depression or Clinically Relevant Depressive Symptoms and on Change in Mood Scores: A Randomized Clinical Trial. Jama, 324(5), 471-480. https://doi.org/10.1001/jama.2020.10224

35. Westreich, D., & Greenland, S. (2013). The table 2 fallacy: presenting and interpreting confounder and modifier coefficients. American Journal of Epidemiology, 177(4), 292–298. <https://doi.org/10.1093/aje/kws412>

36. VanderWeele T. J. (2016). Mediation Analysis: A Practitioner's Guide. Annual Review of Public Health, 37, 17–32. https://doi.org/10.1146/annurev-publhealth-032315-021402
